# Progress and challenges in the computational prediction of gene function using networks

**DOI:** 10.12688/f1000research.1-14.v1

**Published:** 2012-09-07

**Authors:** Paul Pavlidis, Jesse Gillis

**Affiliations:** 1 Centre for High-Throughput Biology and Department of Psychiatry, University of British Columbia, Vancouver, V6T1Z4, Canada; 2 Stanley Institute for Cognitive Genomics, Cold Spring Harbor Laboratory, Woodbury, NY, 11797, USA

## Abstract

In this opinion piece, we attempt to unify recent arguments we have made that serious confounds affect the use of network data to predict and characterize gene function. The development of computational approaches to determine gene function is a major strand of computational genomics research. However, progress beyond using BLAST to transfer annotations has been surprisingly slow. We have previously argued that a large part of the reported success in using "guilt by association" in network data is due to the tendency of methods to simply assign new functions to already well-annotated genes. While such predictions will tend to be correct, they are generic; it is true, but not very helpful, that a gene with many functions is more likely to have any function. We have also presented evidence that much of the remaining performance in cross-validation cannot be usefully generalized to new predictions, making progressive improvement in analysis difficult to engineer. Here we summarize our findings about how these problems will affect network analysis, discuss some ongoing responses within the field to these issues, and consolidate some recommendations and speculation, which we hope will modestly increase the reliability and specificity of gene function prediction.

## Background

A central challenge in genomics is the determination of gene function. As data sets characterizing genes grow in size and complexity, it seems self-evident that computation can assist in inference as to gene function. However, despite extensive work over the past decade, computational determination of gene function has made only uncertain progress. With the important exception of the use of sequence similarity, it is still uncommon for experimental researchers to use computational gene function prediction methods as a starting point in a study. Instead, such methods seem more commonly used by computational methods developers, and by experimentalists who are seeking post-experiment interpretation of a result (with the attendant danger of confabulation). While there are exceptions, in the past few years we have been struck by the gap between the proliferation of function prediction methods and the rate of discovery of gene function, particularly for genes which are not already well characterized, despite the enormous increase in the amount of available data.

In a recent series of papers ^1– 3^, we lay out a case that much research for the analysis of gene function from network-like data (using Guilt By Association; GBA) is based on somewhat shaky premises. The guilt by association principle states that genes with similar functions will tend to be associated (or possess similar properties), allowing previously unknown functions of a gene to be statistically inferred given some prior knowledge about other genes, and association data. Our studies were specifically motivated by challenges we encountered in applying GBA to real-life gene function prediction problems. We uncovered a range of underlying biases that caused the results of GBA to be misleading, which turned out to be pervasive yet previously undocumented. We believe the biases we have described are part of the reason for the relatively limited success of computational GBA. In those papers we described control experiments and other considerations that, we hoped, would help the field move forward. At the same time, we recognized that the challenges were profound and could not identify a one-size-fits-all solution that avoids the biases yet still yields demonstrable and useful gene function prediction performance. In the months since our publications appeared, we have had the opportunity to discuss our findings with many colleagues, and realized that there was a need to summarize and unify the arguments we made. In this commentary we begin by briefly reviewing the key findings of our studies, expand on some points, and address some of the issues that have come up in the meantime. Our aim is to spark further discussion and we hope take advantage of this venue to provide updates and additions.

In discussing GBA, we are specifically referring to the use of the inference as a computational tool to predict gene function from large data sets. As a biologically-motivated principle used to infer gene function on a gene-by-gene basis, GBA is not controversial and long predates the advent of “gene network analysis” approaches. In addition to its potential use for predicting gene function, the GBA principle is also used as “independent” verification of experimentally derived target gene groups or data. In these cases, the ability of an algorithm to learn a group of genes using network data is taken to provide some measure of confidence that the gene set (or possibly network data) has some functional meaning. Indeed, this application is probably more common than the use of GBA for making “de novo” predictions.

To be more concrete about what we mean by GBA, we are concerned with a large class of computational approaches aimed at predicting gene function, which all take as input four things:

1. A set of **candidate genes**, which may be all genes in the genome or a more focused set such as those in a candidate genetic locus. The latter case is often referred to as the “disease gene candidate prioritization” task ^4^ but the distinction is not important for our discussion.

2. One or more **target gene groups** of interest typically defined around a function, such as “synaptic plasticity”, “involved in breast cancer” or “required for the stress response”. We wish to use GBA to assign one or more of the candidate genes to the target gene group. From the point of view of the candidates, GBA assigns a novel target group membership (function) to a particular gene. Operationally, a target group is defined by the set of genes which are already known to have the given property such as membership in a Gene Ontology (GO) group. A target is used even in approaches lacking an explicit target group: we want to be able to act on what we learn, so it has to fit into some pre-existing scheme. Thus while a sequence similarity search does not require a target gene group, interpretation of the results uses such information.

3. **Data** that contains information about associations or similarities among the target and candidate genes. These data are often represented or thought of as a network, but this is not necessarily explicit. Our studies relied largely on coexpression, protein interactions, and genetic interactions, but we also performed experiments with “networks” constructed from sequence patterns, phylogenetic profiles, and phenotype and disease association profiles. Though we use the term “network” it is important to distinguish between this type of abstract network used for inference, and gene networks that represent physiochemical interactions in the cell, though the line between these can be fuzzy.

4. **An algorithm** for transferring functional labels from the target genes to the previously unlabeled candidate genes.

In this article, we use the term “GBA” to refer to the combination of these four factors, not to any one of them. The output of GBA, for a given target gene group (function) is usually a ranking of the candidate genes, where the genes ranked most highly are those which the algorithm predicts are most likely to belong to the target group, given the data. Many (perhaps most) studies undertake evaluations using the Gene Ontology ^5^ as targets under a cross-validation scheme. Performance is typically evaluated using the area under ROC curves, or precision-recall curves. Some studies undertake experimental validation of a subset of “novel” predictions, often focusing on a particular phenotype or function of interest.

In a previous study ^2^, we show that algorithms that use “label propagation” or related approaches can be replaced by methods that, given a sparse network, first propagate edges (so that indirect edges become lower-weight edges in the network), and use a “simple guilt by association” method (neighbor voting). For coexpression data, our data indicate that if possible the original kernel (coexpression matrix) should be used, rather than a sparsified representation. We also showed that coexpression data behaves much like protein interaction data when enough data is combined.

In another study ^1^, we analyzed the effects of bias in the prevalence of genes across target gene sets. By prevalence we mean the number of target gene sets a gene belongs to; this can be thought of as a type of gene multifunctionality. We show that a list of genes ordered by prevalence in GO (and other schemes that might be viewed as alternatives such as KEGG) performs comparably or better to real machine learning algorithms over many prediction tasks, despite lacking any specificity to the particular learning task. In another paper we provide anecdotal evidence that such effects are likely to be at play in disease candidate gene prioritization ^6^. Further, node degree in many networks is correlated with the number of GO terms a gene has, so that much of the performance as evaluated with ROC curves can be explained by algorithms simply assigning all functions to high-degree nodes. We showed that this problem is not readily fixed by various node-degree weighting schemes including filtering out high-node degree genes.

In our most recent work ^3^, we assessed the use of GBA within network data and show that a large fraction of the apparent performance not explained by pure node degree effects are due to the impact of a very small number of edges in the networks, from which it is very difficult to glean generalizable performance. The exception were protein complexes, which display a clique-like structure in many networks, but again it is impossible to generalize from such patterns to make new (non-trivial) predictions.

We realize, and tried to document ^1– 3^ that many researchers in the field have at least a vague sense that something is wrong. In that sense, what we are saying is not news. Our contribution is that we have attempted to document precise explanations for problems which have gone unnoticed for a long time. In the following sections we first summarize the problems we see with GBA, go on to describe why the problems are difficult to fix; provide some suggestions for best practices; and finally close with some speculation.

## A few problems with GBA

**Prior knowledge about gene function is very biased toward well studied genes**. One of our most important claims is that the Gene Ontology (or any of its relatives, which encompasses most such schemes) aligns to the data in ways that are not helpful ^2, 3^. It may be that our collective human-constructed “ontology” of gene function, as powerful as it has been at organizing information, just doesn’t have a sufficiently general relationship with biology as we measure it in the lab. This means that the tasks we are able to learn are so strongly biased by relatively uninteresting interactions between data and the target that more biologically specific signals are probably being missed. In other words, it may be a case where true-but-uninteresting prediction is all but impossible to avoid or improve upon. An interesting side effect of thinking that GO ( *et al.*) is not the ideal target is that the “best GBA method” (by some currently unknown standard) might actually perform badly on GO. Evaluation of such a method (if it exists), other than by exhaustive experimental validation, is currently impossible. We also caution that some of our other points are confounded by our own reliance on existing schemes (including GO), so we leave the possibility open that there is a leap waiting to be made.

**“Good” predictions tend to be generic predictions**. Our results imply that many predictions will be generic ^2^ so the most likely candidate genes will tend to be a gene that has numerous other functions – whether this is already known or not. The gene that is predicted to be involved in muscle development might also be involved in twenty other processes. It is clearly of interest to make predictions that are functionally specific, or at least to know how specific they are. If one is to claim that a GBA approach is “good”, one of the criteria might be that the gene doesn’t have functions that *weren’t* predicted. Ideally in a validation, one should show that disruption of the gene does not affect other functions that were not predicted, thus providing some measure of specificity. We recognize that our suggestion of additional costly and difficult control experiments is unlikely to be popular. However, we fear that the current situation feeds a vicious cycle where GBA continues to seem to work (somewhat) in a way that is misleading to computational method developers and also driving even stronger biases in biological knowledge.

**High performance in cross-validation doesn’t help us find what “works”**. In most studies, cross-validation is used to estimate which functions are learnable. The hope is that that high cross-validation performance for a particular function will generalize to novel predictions for that function, and therefore will hold up in experimental validation. However, our results indicate that while there are “good” pieces of information in network data, the presence of one such piece of information for a given function does not imply the presence of more to be discovered ^3^. This graininess in network information (or lack of “systemic” functional encoding) may mean that a limited number of experimental validations do not validate a method as a *general*-purpose way to determine function. We see it as a substantial challenge to assess what works. For example, how do we define data that is “good” for GBA in a way that will generalize to novel predictions rather than simply tell us “what worked, worked” (maybe only that one time).

**Unbiased data is desirable but (probably) non-existent**. One way to avoid some problems is to only use data from experiments which analyze all genes in the genome at once. This is not always possible, and even when it is, there are still often biases towards (for example) genes which are conserved, have readily cloneable nucleotide sequences, are expressed at readily detectable levels, have well-annotated gene structures, have immunogenic products (so they yield good antibodies), are suppressible by siRNA, are non-essential, and so on. We refer to these as biases because any correlation between the prevalence of genes in a dataset and their prevalence within GO (number of annotations) will generate apparently effective GBA in which a subset of genes or connection will dominate results with either little specificity or little generalizability. Such factors should be at least considered as confounds, and representation or prevalence discrepancies should ideally held as constant as possible when data sets are integrated. We feel the terminology “unbiased” should be avoided except in relative terms, or that at least it should be clearly stated which biases are being referred to.

**Even if GBA works, it may not work well enough for experimental follow-up**. It is very unclear what degree of precision is obtained in “real-life” applications of GBA in which experimental validation is performed. Obviously it varies, but our impression is that it is much worse than what biologists would usually refer to as “statistically significant” – that is, 95% confidence in a single prediction. In general, significance from computational methods arises from aggregate performance of groups of genes, but this is less useful in providing (potentially costly) experimental targets. In a recent study, a false discovery rate of 87% was reported ^7^. Another study reported a more impressive but still high false discovery rate of 44% ^8^. Clearly such rates are sufficient to be of use (a bona fide new drug target is worth a lot of trouble), but in our experience they are also low enough to discourage many biologists from routine follow-up of computational predictions. We note that neither of the studies just mentioned were formal assessments in the sense that the benchmark was not held in escrow by a third party. In addition, they only validate predictions for a couple of functions, so it may be risky to generalize to other functions. Additional assessments that take a formal approach would help advance the field, but designing such challenges and evaluation is not trivial.

## Warning against easy-fixes

**It is easy to hide the problems**. Many of the problematic aspects of an analysis are most easily observed due to their side-effects. For example, one of our observations is that, according to many commonly used metrics, a sizeable fraction of performance can be explained by simply predicting high node degree genes often (because high node degree genes are often multifunctional). It is trivial to remove the appearance of this problem, particularly by alterations in the metric or network (simply add random connections to make node degrees equivalent). However, the underlying problem (overly generic results contribute to performance) could remain with the issue simply having been hidden from view. Similarly, some problems are made clearer when using ROC while others are revealed by using precision-recall (ROC being susceptible to overly generic predictions, while precision-recall is affected by non-generalizable one-off observations). Unfortunately, one possibility is that some algorithm’s bad behavior will simply become harder to observe in the face of better characterization of the problems.

**Improving GBA cannot be done by enforcing a better match between data and targets**. It may be tempting to consider cross-validation performance as the most important metric of a method’s utility, but this is a very dangerous assumption (as is well-appreciated in the machine learning field). In one version of this point of view, poor cross-validation performance is viewed as meaning that the data are not good enough, and data should therefore by chosen based on what works best. Thus some researchers have proposed to restructure or re-weight data that better matches the gold standard (e.g. GO) ^9– 11^. A closely related tactic is using the Gene Ontology as a data source ^12^. The potential for overfitting/overtraining and logical circularity encompassed by such approaches should be abundantly clear. In particular, selecting the best performing datasets may tend to increase the biases which do, indeed, yield high (but useless) performance. Similarly, one might consider changing the ontologies so that they better match guilt by association results. That is, instead of changing the data change the target groups (thus far we have only heard this raised as a possible strategy). We can only think of this as “moving the goal posts” and extremely risky if all parties are not aware of the effect this would have on predictions. We think the separation of GBA as a top-down principle (in algorithms) must be maintained from bottom-up observations (from which derive ontologies and data). Otherwise GBA’s potential for making new discoveries will be crippled.

## Suggestions

**We shouldn’t confound discussions about performance with novel or non-standard metrics**. As important as settling on a method is settling on an evaluation metric. Everybody’s approach works best by some metric. Introducing novel or *ad hoc* metrics to go with a novel prediction method simply muddies comparisons. In our experience, biologists are most interested in precision, less interested in recall, and generally not receiver operating characteristics (whether they know the jargon or not). Reports of performance should be corrected for multiple testing if multiple metrics are used.

**Ignoring “low quality” GO evidence codes is not as straightforward as it seems**. It is common to exclude “IEA” (inferred by electronic annotation) GO annotations from computational analyses. The rationale usually given is that IEA annotations are unreliable (this is somewhat ironic since what GBA methods are trying to do is provide IEA annotations). In any case, hiding such annotations from training and test data can lead to the possibility that one can merely reconstruct them in a manner that, in effect, the GO consortium has already done (predominantly by leveraging sequence similarity). In addition, many high-quality annotations in GO started out as “IEA” and were specifically targeted for further manual curation. This throws into question the purity of “rollback” validations that consider only manual annotations from before a certain time limit during training, and test on more recent annotations. On the balance it is probably best to include IEA annotations throughout and that to be of real value, function predictions should go beyond what GO is already providing.

**Function prediction should not *only* be information retrieval**. We wish to make a distinction among different types of “automated annotation”. There are many pieces of functional data in the biomedical literature that have not been converted to (for example) GO annotations, so they are difficult to access. It is an interesting task to attempt to mine such information from the literature directly or indirectly. However, this is not prediction, but information retrieval. This basically ensures that in some sense the most confident predictions made will tend not to be novel, but capture information that is already known. We feel this task is different from cases where the function prediction is “de novo”, without using existing functional annotation. We realize there is no firm line between a supposed piece of information retrieval and a prediction, and it is an interesting question whether it is even possible to make de novo predictions (or even what that would mean in a pure sense), because information has the potential to leak between data types in insidious ways. For example, expression microarrays are biased against the representation of poorly-characterized genes.

**Assessments of performance should use well-chosen priors**. A theme that has emerged is the urgency to more carefully assess null performance in the evaluation of gene function prediction. In particular, we should be determining the performance of algorithm learning from the data relative to the performance using well-chosen priors. This is rather obvious because methods can exploit the choice of prior, but less obvious because current methods of validation tend to miss this point. We should be thinking of ways to cheat at prediction (yielding apparently good but useless performance) and taking this as the baseline upon which we must improve. Usually if machine learning algorithms can cheat in this sense, they will (without any collusion on the part of their designers). Information leakage between training and test data, despite the best intentions, is very hard to avoid. We showed that current approaches inadvertently strongly exploit the choice of prior, and this makes prediction methods much less useful than is commonly claimed. This decrease in utility comes both from limitations in functional specificity (many predictions will be very general such as “growth”), prediction specificity (many predictions will just be wrong), and “rich get richer” effects (genes that have lots of functions will be proposed to have more). We urge investigators to include assessments of the impact of node degree and of critical edges on their predictions.

**Data drives performance more than algorithms**. We presented evidence that the variance in apparent prediction performance among approaches is largely explained by differences in the data, not the algorithm. Differences in algorithms play a relatively minor role. This is a banal observation in machine learning at large, but in gene function prediction we are only learning this lesson now. Besides the experiments presented in ^2^, we point to the close performance of different methods in the Mousefunc assessment ^13^. A recent evaluation of gene network inference methods (which largely rely on guilt by association), the DREAM5 challenge, further supports our claim. Simple “off-the-shelf” methods performed competitively with other more complex approaches, and the best methods were distinguished by which of the available data were used ^14^. This is not to say that one can’t do badly at such an assessment, but that the upper bound is rapidly reached by a reasonably good algorithm. We feel it is unlikely that substantial progress will be made by working on developing new algorithms, and that the focus should be on the data. This is a sufficiently important issue that in our opinion, it would be interesting to consider basing critical assessments on a single method (or aggregate of methods), and letting data vary as desired.

**Context specificity of data can help**. One of the factors that cause functional predictions to be generic is that they often use data that combined numerous experimental conditions; protein interaction networks constructed by aggregated data sources are chief among these. It would be helpful to be able to show that a function prediction is appropriately context-specific. For this reason, we predict that analyses that use context-specific data will increase. One example of data that is in principle context-specific is gene co-expression. Co-expression occupies an unusual place in high-throughput analyses. For researchers focused on particular biological problems, it is a relatively easy way of obtaining context specific data. In contrast, to researchers focused on computational methodologies it is often regarded as a noisy data source which poorly recapitulates known biological function as catalogued in GO. However, many of our results support the view that co-expression data is much more difficult for algorithms to “cheat” on, since it less specifically captures literature biases. Other forms of data specificity (e.g., different environments or phenotypes used for assaying genetic interactions) are also strongly desirable to determine the robustness of results. Of course, even with such data it remains important to perform controls for specificity (e.g., constructing control networks, checking control gene sets that matched with respect to properties that allow cheating).

## Speculation

**Biases affect network analyses besides functional prediction**. Because we suspect most variance in GBA performance is data driven, we believe the biases we have discussed in GBA are also partially data driven. That is, they are not just a feature of the GBA method, but of the data. An example of another type of analysis is predicting interactions themselves, as opposed to functions. We strongly doubt whether “association by guilt” is fundamentally different from guilt by association. For example, we would expect some predicted connections to be trivial (e.g., interacting promiscuous proteins) with protein complexes again being a special case (i.e., filling in a few missing connections from a fully connected sub-graph). We likewise suspect that our finding that many function prediction algorithms act as if they are reconstructing filtered values through indirect connectivity ^1^ may apply to predicting connectivity itself; perhaps the best predicted connections are simply those that were “nearly known” to begin with. Another topic we have not considered extensively is unsupervised approaches, exemplified by the WGCNA coexpression graph clustering method ^15^. These methods are typically combined with looking for clusters that are enriched for certain gene functions. We consider it likely that such approaches are subject to many of the same problems as the supervised methods, including biases toward highly-annotated and highly-connected genes.

**GO is here to stay**. As outlined above, it is unlikely that we will have a true replacement for GO any time soon, in part because there is no clear idea of what it would be based upon. Currently available alternatives to GO (as a source of functional gene sets) such as KEGG mainly resemble it in terms of annotation biases. In the meantime, the appropriate use of GO is not at all settled (e.g., ^16^) and we expect that awareness of the limitations of GO as a target for function analysis will have an increasing impact.

## Conclusions

Despite all the problems and limitations we describe, we believe there are still good reasons to be optimistic about the future of GBA. While claims of being able to predict function globally should be treated with greater skepticism, focused analyses will likely continue to pay gradual dividends. Similarly, while validation of globally applicable methods may succeed in focused projects, the methods themselves are very unlikely to be universally successful and claims within the literature should be tempered by more detailed and explicit discussion of exact limitations.
