# Supplementary material for: Progress and challenges in the computational prediction of gene function using networks
Source: F1000Res. 2012 Sep 7;1:14. [Version 1] doi: 10.12688/f1000research.1-14.v1 (PMC3782350; doi:10.12688/f1000research.1-14.v1)
Supplement: Peer review history

# Peer review: Referee response for version 1

**Published:** 2012-09-25

**Authors:** Jonathan D Wren

**Affiliations:** 1 Biochemistry and Molecular Biology Department, Oklahoma Medical Research Foundation, Oklahoma City, OK, USA

The authors synthesize, summarize and clarify their prior work and observations on predicting gene function using linked (networked) data to infer properties of unannotated/uncharacterized nodes on the basis of their surrounding nodes.

Their observations and cautionary notes are important for people attempting to infer function using this “guilt by association” (GBA) approach, or, as it might alternatively be called in machine learning, the multi-label classification problem.

Some of the problems brought up, though, may not be readily surmountable, such as the bias in annotation whereby some genes are extremely well annotated and some categories/labels are far more frequent than others. Insofar as science proceeds by building upon prior observations, the use of existing gene knowledge to infer new knowledge will only be able to frame predicted gene function in terms of previously characterized functions. e.g., an uncharacterized gene predicted to affect DNA repair cannot have its precise role/purpose in DNA repair predicted, but rather can only be associated with previously characterized DNA repair phenotypes (e.g., non-homologous end joining, double-strand break repair, etc). Additionally, its not clear what the gap is between how frequent a phenotype (e.g., angiogenesis) is empirically and how frequent it is among predicted functions. This is an important limitation of the method, but insofar as existing data can be used to guide experimentation, it is still useful as long as one takes into account the authors’ caution that “good” algorithmic predictions tend to be generic.

In this reviewer’s experience, and with his own particular approach to the problem, the use of GBA has led to successful characterizations of several genes (PMIDs: 22187488, 21868574, 19646878, and one recently accepted for publication in Neurosurgery). This does not invalidate the authors’ points, which are well taken by this reviewer, nor is it proof of efficacy. However, it is offered as non-systematic evidence that GBA has been useful/successful in guiding experimentation. Systematic approaches and appropriate negative controls are important, as the authors suggest, and need to be done. The field is relatively young, however, and I think the observations by Pavlidis and Gillis discussed here are important to help separate the promise and the peril of research in this area, as I believe the GBA approach (in principle, not necessarily in practice) holds the potential to aid experimental science. Their observations are important to consider in terms of interpreting prediction results and guiding future research in predicting gene function.

I have read this submission. I believe that I have an appropriate level of expertise to confirm that it is of an acceptable scientific standard.

# Peer review: Referee response for version 1

**Published:** 2012-09-11

**Authors:** Yves Moreau

**Affiliations:** 1 ESAT/SCD, K.U. Leuven, Leuven, Belgium

This article is an opinion piece based on previous peer-reviewed work of the authors. As such it appears of appropriate quality.

I have read this submission. I believe that I have an appropriate level of expertise to confirm that it is of an acceptable scientific standard.
